# A novel approach to assess clinical competence of postgraduate year 1 surgery residents

**DOI:** 10.1080/10872981.2017.1342523

**Published:** 2017-07-03

**Authors:** Xin Qi, Lian Ding, Wei Zhai, Qiang Li, Yan Li, Haichao Li, Bing Wen

**Affiliations:** ^a^ Department of Plastic Surgery & Burns, Peking University First Hospital, Beijing, People’s Republic of China; ^b^ Academy for Advanced Interdisciplinary Studies, Peking University, Beijing, People’s Republic of China; ^c^ Department of Cardiac Surgery, Peking University First Hospital, Beijing, People’s Republic of China; ^d^ Department of Respiratory Medicine, Peking University First Hospital, Beijing, People’s Republic of China

**Keywords:** Standardized training of residents, OSCE, irregular wound, debridement, suture

## Abstract

**Background**: An increased demand for accountability and transparency in medicine have initiated a shift toward a more objective and standardized approach for postgraduate medical training.

**Objective:** To develop and evaluate an objective method to assess clinical competence of postgraduate year 1 surgery residents.

**Design**: Thirty-one postgraduate year 1 surgery residents, who had been trained in the Surgical School of Peking University First Hospital for one year, participated in an objective structured clinical examination as a final assessment of their clinical competence. A test station of irregular wound repair (debridement and suture) was specially designed to test the residents’ surgical integrative competence in a complex-trauma treatment procedure. A modified global rating scale, in combination with wound area measurement, was applied to evaluate residents’ surgical performance. The validity of the subjective global rating scale was evaluated by the objective measurement results from the software.

**Results**: The global rating scale score had no obvious correlation with the area of the removed tissue and the residual wound area after the suture. There was significant difference in the debridement time and the residual wound area between 0–3 and >3 total stitches. There were significant differences in the area of the removed tissue between 0 and 1–2 grey stitches and 0 and 3–4 grey stitches, and in the residual wound area after suture between 0 and 3–4 grey stitches and 1–2 and 3–4 grey stitches.

**Conclusions**: An irregular wound repair procedure could be an effective method to assess the integrative competence of surgery residents. The training for surgical thinking in the early stage of junior residents needs to be strengthened. The entire measurement process was more complex and time-consuming than expected. The possibility of measurement by simply counting the numbers of the key spots might be explored in the future.

**Abbreviations:** ACS/APDS American College of Surgeons/Association of Program Directors in Surgery; GRS Global rating scale; LSD-T Least significant difference-test; OSATS Objective structured assessment of technical skills; OSCE Objective structured clinical examination; PBT Proficiency based training; PGY1 Postgraduate Year 1

## Background

Since the last century, William Halsted’s classical model has been developed and used in surgical education. This Halstedian method [1] of surgical training is often exemplified as the ‘see one, do one, teach one’ approach to training, resembling a master-apprentice training mode. It has been considered that clinical competence of surgery residents can be enhanced by (1) increased exposure to surgical procedures (2), bedside care of patients (3), copying from role models, and (4) receiving verbal instructions from expert surgeons [2,3]. Although this surgical training model continues to have a strong impact on surgical education, it has been criticized for its high investment in training time and teaching staff resources [4]. The current medical education environment in China does not allow ample opportunities for each resident to receive adequate training in the operating theater. Therefore, disparity in training time and opportunities may affect residents’ clinical competence and, in turn, patient safety. These trends, in combination with an increased demand for accountability and transparency in medicine, have initiated a shift toward a more objective and standardized approach for postgraduate medical training. As part of a national initiative to reform postgraduate medical training, Peking University First Hospital started in 2014 the first surgical school in China, aimed at formalizing and standardizing training for postgraduate surgery residents from the first residency year.

In a standardized training program, there is a strong need for educational tools to provide objective assessments to evaluate predefined competency goals [5]. The current competence evaluation tools are mostly scales or checklists that assess residents’ technical skills based on the evaluator’s judgment of surgical performances. The faults or imperfections in surgical procedures are corrected using the evaluators’ verbal or written feedback. However, whether an objective assessment of procedure correctness can guarantee the desired outcome is debatable. Theoretically, proficiency-based training (PBT) involves deliberate practice and immediate feedback. It emphasizes the value of practice. Repeated practice is assumed to help the trainees develop clinical skills transferability required in real-life complex medical situations. PBT enables trainees to understand the reason for, and the manner in which to carry out, surgical procedures. However, whether students can effectively transfer these skills to real-life complex clinical occasions is still doubtful. Moreover, evaluation of residents’ skill transferability may pose a great challenge for training program designers.

Surgical clinical competence was defined as the integration of technical and non-technical skills with clinical thinking, which mainly involves the choice and matching of surgical techniques. To make an objective evaluation of the surgical integrative competence of residents after a year’s training in our surgical school, we designed an examination of irregular wound repair (debridement and suture) as a test station for an objective structured clinical examination (OSCE). This was to evaluate their capability in handling complex wound cases. We also measured the debridement time, the area of the repaired wound, and other objective indicators, in what is known as an outcome-based assessment approach, comparing it to a modified Global Rating Scale (GRS) score. We try to estimate if these objective indicators are better than the GRS at assessing clinical competence.

## Methods

### Participants

Thirty-one surgical PGY1 (Postgraduate Year 1) residents (30 males, 1 female) in our surgical school volunteered to participate in the study. Their subspecialties were already chosen before entering into our program, and these included general surgery (10 participants), urology (eight participants), oncology (five participants), orthopedics (three participants), neurosurgery (two participants), cardiac surgery (two participants), and plastic surgery (one participant). All the participants were right-handed.

Their average age was 24.94 ± 1.67 years. The average time of surgical rotation was 9.48 ± 1.79 months.

### Research design and procedure

**Figure 1.**
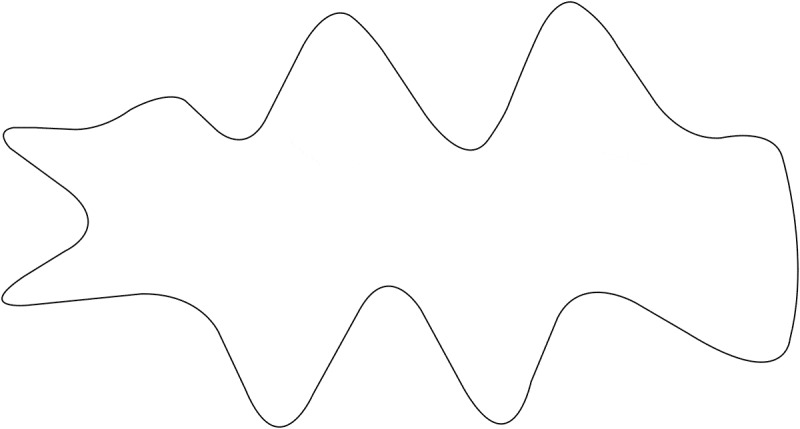
Irregular wound mold design.

Using the same mold, irregular wounds were drawn on cadaveric pork belly skin. The tissue within the shape drawn was regarded as the necrotic part, involving deep fascia (see Figure 1). Necrectomy and primary suture were to be completed within 15 minutes.

The area of the irregular wound was 33 cm^2^. After debridement, the maximum width of the defect was 3.8 cm, and the maximum length was 10.0 cm.

The participants were asked to do the resection and primary suture procedures. For resection, they were instructed to excise the necrotic tissue accurately and completely, but at the same time, to preserve and protect as much normal tissue as possible to reduce repair difficulty. For the suture procedure, they were expected to apply ‘tension-free’ wound closure to the resected area and perform deformities repair (like dog-ear).

Currently, Objective Structured Assessment of Technical Skills (OSATS) is one of the most frequently used and widely accepted observational instruments, considered the golden standard for assessment of surgical skills [6]. The original OSATS consists of a three-part assessment form, including a task-specific checklist, a GRS, and a pass/fail judgment [7]. The GRS is considered superior to task-specific checklists in terms of reliability and validity [8,9]. Therefore, we adopted only the GRS to evaluate the surgical skills of the PGY1 residents. The scale was slightly modified to meet the needs of our tests. We deleted the domains of little relevance to the required skills and added the domains most pertaining to irregular wound repair procedures. Consequently, the modified scale included the following domains: ‘Maintaining a Sterile Field,’ ‘Knowledge and Handling of Instrument,’ ‘Quality of Excision,’ ‘Quality of Debridement,’ ‘Dissociation of Subcutaneous Tissue,’ and ‘Quality of Suturing and Knots.’ Furthermore, the 5-point Likert scale was replaced by a 7-point Likert scale with scores ranging from 4 to 10 [5,10]. The total score of the modified scale was 60 points.

**Figure 2.**
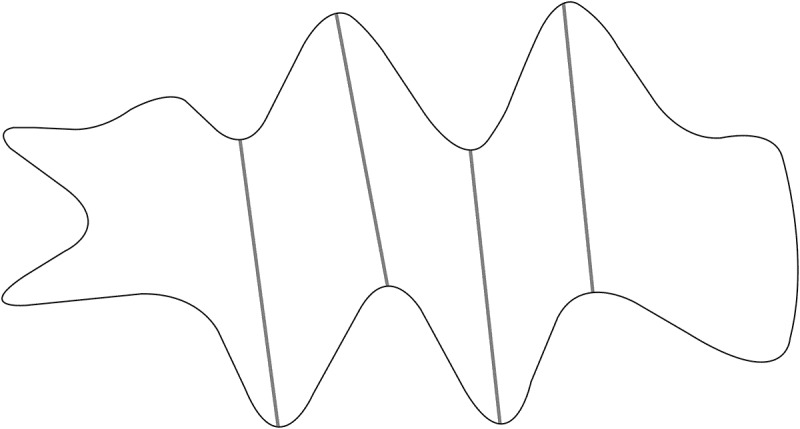
The schematic diagram of objective scoring criteria.

**Figure 3.**
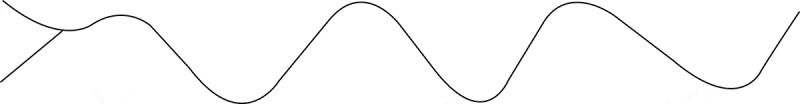
The schematic diagram of the recommended result.

Participants were expected to first suture the key spots marked by grey stitches in Figure 2 to achieve primary closure of the wound. If they could successfully finish the grey stitches within the limited testing time, they were considered to have holistic surgical thinking competence prior to the operation, effectively judging the wound and weighing the techniques rather than carrying out the operation aimlessly. The recommended ideal outcome is shown in Figure 3.

Three raters were trained prior to the test to ensure consistency in their observation and scoring criteria. Raters were not blind to the training background of the residents. They independently scored each participant’s performance right at the examination station using the scales described above. The GRS score was calculated by averaging the raters’ scores on the global rating scale. The number of completed grey stitches and the total number of stitches were recorded as well.

In order to assess the actual efficiency of the PGY1 residents’ operation, we measured the debridement time, the area of the removed tissue, and the residual wound area at the end of the examination by automated type-based wound surface area assessment software [11]. Since the cadaveric pork belly skin had no elastic retraction, we considered the area of the removed tissue to represent the area of the wound after debridement.

## Data analysis

All results were expressed as means and standard deviation in the form of means ±SD. The data were statistically analyzed using the t-test, least significant difference-test (LSD-T), and Kolmogorov-Smirnov statistic and linear correlation, using SPSS 22.0 software (IBM Corporation, USA). A *p*-value of less than 0.05 was regarded as statistically significant.

## Results

### Assessments and scores

In the examination, 30 residents succeeded in excising the necrotic parts of the irregular wound, whereas only 1 resident was not able to complete the debridement. To the surprise of the faculty, there were 2 residents almost precisely completing the primary suture procedure in accordance with our recommended suture outcome.

The average score of GRS of 31 participants was 36.91 ± 3.29.

### Correlation analysis

Correlation analysis was run between the GRS score and the objective efficacy measurement. The results are as shown in Table 1 (excluding the score of the resident who did not complete the debridement). It was found that the global rating scale score was not significantly correlated to debridement time, the removed tissue area, and residual wound area after suture.

**Table 1.** Correlation analysis between global rating scale score and efficiency measurement.

	Global rating scale	
	Pearson correlation coefficient	*p*-value
Debridement time(s)	–0.28	0.139
Area of the removed tissue (cm^2^)	–0.11	0.572
Residual wound area after suture (cm^2^)	–0.31	0.101

*n* = 30, *p < 0.05, **p < 0.01

### Comparison of efficiency measurements

**Figure 4.**
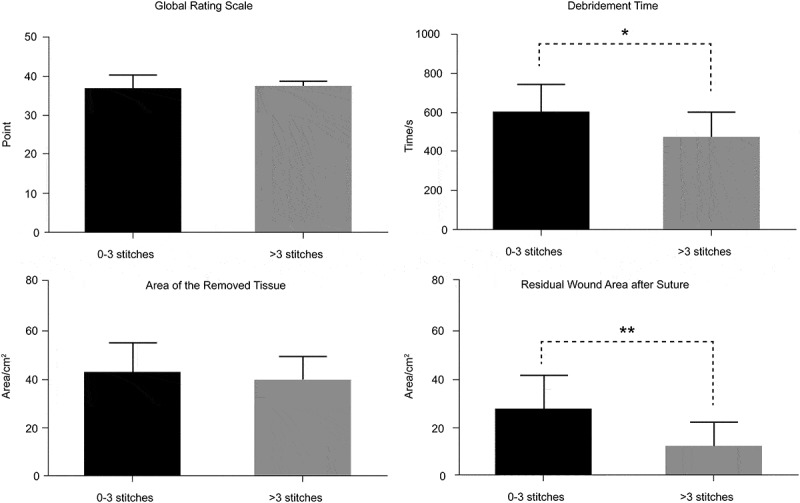
Comparison of the efficiency measurement between 0–3 and >3 stitches.

The median of the total number of stitches is 3. According to the median, we divided them into two groups (0–3 and >3 stitches). There were significant differences in the debridement time and the residual wound area between the two groups (Table 2, Figure 4).

**Table 2.** Comparison of the efficiency measurement between 0–3 and >3 stitches.

	0–3 stitchesn = 16	>3 stitchesn = 14	*t*	*P*
Global rating scale	36.20	37.79	−1.358	0.155
Debridement time(s)	598.00	467.29	2.507	0.018^a^
Area of the removed tissue (cm^2^)	42.83	39.51	0.814	0.422
Residual wound area after suture (cm^2^)	27.56	12.28	3.315	0.003^aa^

^a^*p *< 0.05, **p < 0.01

**Figure 5.**
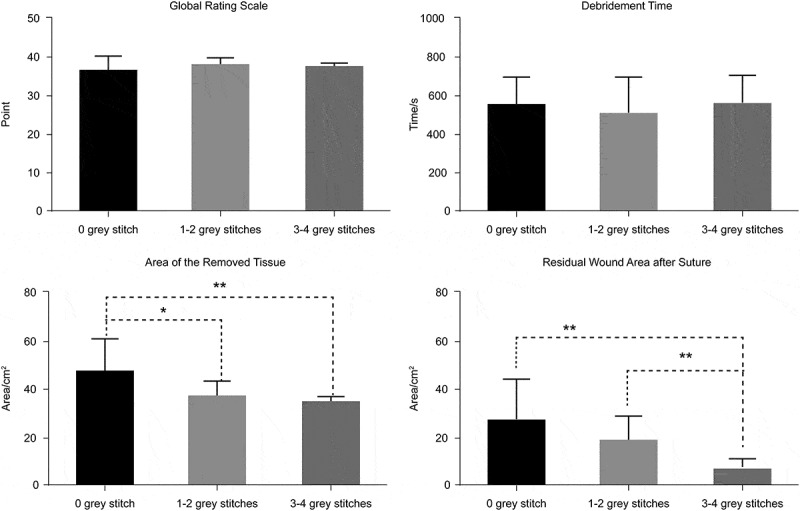
Comparison of the efficiency measurement between the different grey stitches.

Efficiency measurements using the different number of grey stitches are as seen in Table 3 and Figure 5. There were significant differences in the area of the removed tissue between 0 and 1–2 grey stitches and 0 and 3–4 grey stitches, and in the residual wound area after suture between 0 and 3–4 grey stitches and 1–2 and 3–4 grey stitches.

**Table 3.** Comparison of efficiency measurement between different grey stitches.

	0 grey stitchn = 14	1–2 grey stitchesn = 10	3–4 grey stitchesn = 6	^1^P	^2^P	^3^P
Global rating scale	35.93	38.00	37.56	0.169	0.383	0.517
Debridement time(s)	548.7	505.00	563.00	0.523	0.839	0.525
Area of the removed tissue (cm^2^)	47.14	36.81	35.05	0.020*	0.005**	0.428
Residual wound area after suture (cm^2^)	27.2	19.08	6.87	0.176	0.009**	0.009**

P-value between: ^1^P: 0 and 1–2; ^2^P: 0 and 3–4; ^3^P: 1–2 and 3–4

*p < 0.05, **p < 0.01

## Discussion

The standardized training of residents is aimed at providing equal access to residents to master standard knowledge, skills, and attitude. Residents have to first acquire the basic skills in the targeted domain, become proficient through repeated practice, until consequently they can transfer these skills to a variety of clinical situations in an appropriate manner [12]. The novice to expert trajectory is the course from unconscious incompetence to unconscious competence [12], and the currently available PBT can accelerate this transformation. Based on this theory, we hoped to evaluate PGY1s’ unconscious reaction to complex medical events through this novel test approach – a test on an irregular wound repair, which could pose a high demand on residents’ integrative competence. Not surprisingly, we observed that some of the PGY1 residents were sweating and anxious, and began dislocating the sutured area when they encountered complex surgical procedures at the test station. Therefore, it is necessary for the training program designers to simulate to a greater extent the tension and stress of real-life complex clinical practices by creating novel test scenarios, reducing operation time, and increasing test difficulty. Through these practices, residents’ clinical competence and unconscious reaction can be manifested and developed.

Debridement and suture are essential surgical technical skills, which have to be mastered in the American College of Surgeons/Association of Program Directors in Surgery (ACS/APDS) surgery resident skills curriculum [13] for PGY1–2. In China, PGY2 have started to complete their rotation in the emergency department where they encounter different traumas. To prepare PGY1s for their PGY2 training and make an easy evaluation of debridement and suture outcomes, we designed the test station for irregular wound repair (debridement and suture) to assess PGY1s’ integrative competence in handling complex trauma, which integrated the testing of items 1–5 required for mastery in Phase 1 of the curriculum.

Technical skill is unique to surgical practice [14]. The current ‘gold standard’ for the objective assessment of technical skill is OSATS [6], which has been shown by many studies to have sound reliability [15]. OSATS mainly depends on trainers’ direct observation in assessing trainees [16]. The focus of OSATS is on ‘surgical procedure’ and not on ‘surgical products’. It has been constantly argued whether the procedure’s correctness can ensure the validity of the outcome [17,18], which is also a problem to be considered when designing an evaluation scale. Our study results revealed that the GRS had no obvious correlation to the objective measurement of surgical outcomes, which lends further evidence to the argument that procedure-based OSATS evaluation does not guarantee the validity of surgical outcomes. In this study, we adopted automated type-based wound surface area assessment software to measure the size of the removed tissue and residual area of the wound after suture. These measurement results can help us assess the outcome of the operation objectively, reliably, and accurately.

Another important finding of our study is that all teachers can easily retrace the thinking process of PGY1s through the final photos and provide feedback to the residents directly. On completion of the resection and suture procedure, raters can work out how the residents judged the depth and width of the necrotic area of the wound, whether they correctly chose the key suture sites, how they planned their wound repair, and what factors they took into consideration in producing the final surgical outcome. The traditional procedure-based assessment can merely evaluate whether the residents have mastered the necessary knowledge and basic skill points. However, these scattered knowledge points and unsystematic skills training failed to prepare residents to face the challenges of complex clinical practices. Real-life clinical practice involves linking these scattered points in a line and following the correct order; therefore, it requires residents to have the right clinical thinking before practice and achieve their clinical competence. The test can effectively trace their surgical thinking process and assess their clinical competence. Our test results revealed that most PGY1s are mechanical in implementing procedure and deficient in necessary surgical thinking. For example, in the examination, we observed many defects in PGY1s’ procedures. None of the residents dissociated the subcutaneous tissue to reduce wound tension. Some PGY1s cut parts of the muscle to close the wound, achieving ‘closure’ at the cost of ‘depth.’ In unfamiliar situations, PGY1s were not able to extrapolate from what they had learned. These observations suggested that our PGY1s need more training in surgical integrative skill and need to develop their surgical thinking.

Further, we divided the residents into two groups according to the total number of their stitches. With no significant differences in the GRS, the residents differed from the operative outcome. The total number of stitches could effectively distinguish the high- and low-skilled residents. Their proficiency can thus influence the result directly. According to the grey stitches (key spots), we divided them into three groups (0, 1–2, 3–4 grey stitches). 3–4 grey stitches proved that the residents definitely knew how to handle the complex wound. 1–2 grey stitches showed they knew a little or maybe it was a coincidence. Although the debridement time of the 3–4 grey stitches group is longer than that of the others, there is significant difference in the outcome of the residual wound area after suture between the 0 and 3–4 stitches group and the 1–2 and 3–4 grey stitches group. This suggests that proficiency is not sufficient to fill the gap of surgical thinking. However, there are no significant differences in GRS score between different groups. Therefore, we can draw the conclusion that GRS score can only evaluate whether the procedure meets the criterion, but it is not able to judge the surgical outcome. The current surgery resident training program needs to change the practice of overemphasizing proficiency rather than efficiency to enable residents to perform unfamiliar, complex medical practices with competence.

This level of examination is like a ‘mission impossible’ for most PGY1s. However, in clinical practice, they are likely to come across similar situations anytime and anywhere. Therefore, this test can prove to be a great challenge as well as a great benefit for PGY1s to foster precise and prompt surgical thinking in their early stage of resident training. Through constant training and practice, they can transform these ‘mission impossible’ tasks to ‘nothing is impossible’ tasks.

This study also had a few limitations, which are detailed below. Above all, the measurement process is more complex and time-consuming than expected. So the possibility of measurement by simply counting the numbers of the key spots might be explored in the future. Besides, our sample size was small, which limited the generalizability of the results. Independent and closed spaces should be provided to each trainee to avoid interference from one another. A video of their performance should have been recorded [9] for a double-blind evaluation, which could help avoid the subjective preference of raters for particular trainees [19].

## Conclusion

This novel assessment approach has effectively evaluated the surgical outcome of PGY1 residents, and more importantly, it has reflected to some extent their surgical thinking competence. Our test results indicate that residents of the program are deficient in wound judgment, technique selection, and wound management decisions, especially under great time pressure. Therefore, it is suggested that surgical thinking training should be integrated into the early stage of residents’ PBT.

## Declarations

**Ethics approval and consent to participate**

The study was approved by the Peking University First Hospital’s ethics committee, and patient consent was obtained.

## Availability of data and material

The dataset supporting the conclusions of this article is included within the article.
